# P-403. Optimizing diagnostic approach for Ventilator Associated Pneumonia (VAP) in a Pediatric Intensive Care Unit (PICU)

**DOI:** 10.1093/ofid/ofaf695.620

**Published:** 2026-01-11

**Authors:** Helena Brenes-Chacon, Iliana Aguero-Soto, Cristian Perez-Corrales, Christopher Mairena-Acuna, Gabriela Naranjo-Zuniga

**Affiliations:** St. Jude Children's Research Hospital, Germantown, TN; Hospital Nacional de Ninos "Dr. Carlos Saenz Herrera", Cartago, Cartago, Costa Rica; Hospital Nacional de Ninos "Dr. Carlos Saenz Herrera", Cartago, Cartago, Costa Rica; Hospital Nacional de Ninos "Dr. Carlos Saenz Herrera", Cartago, Cartago, Costa Rica; Hospital Nacional Niños, San Jose, San Jose, Costa Rica

## Abstract

**Background:**

Ventilator-Associated Pneumonia (VAP) is a significant cause of morbidity and mortality in critically ill pediatric patients. Accurate diagnosis remains a challenge due to non-specific clinical presentations, inconsistent diagnostic criteria, and limitations in current microbiological and imaging methods. Optimizing the diagnostic approach is essential, so we aimed to measure prescription of bronchoalveolar lavages (BAL) in patients admitted to the pediatric intensive care unit (PICU) as well as consumption of third generation cephalosporins in the only tertiary pediatric hospital in Costa Rica.

**Figure:**
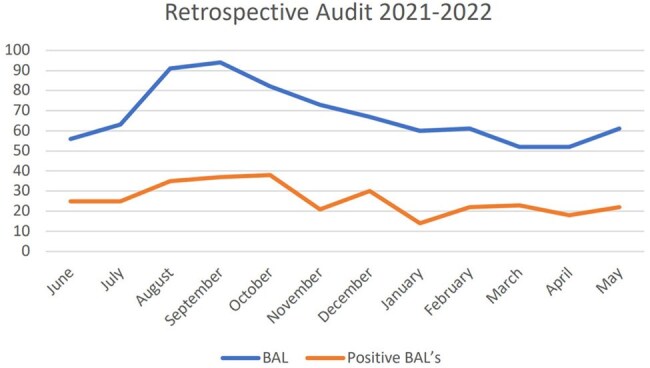
Number of BAL’s taken per month pre-intervention (from June 2021 to May 2022).

**Figure:**
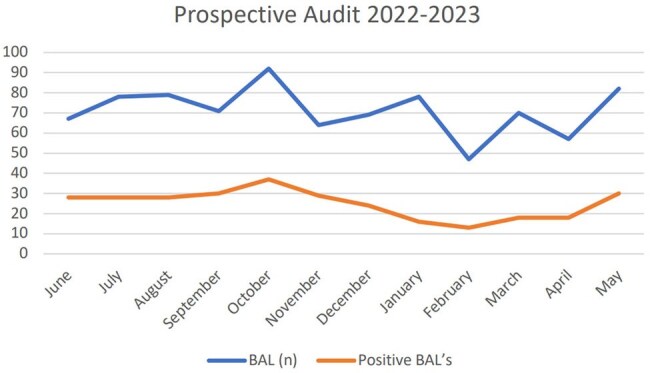
Number of BAL’s taken per month post-intervention (from June 2022 to May 2023).

**Methods:**

A retrospective and prospective audit was conducted analyzing BAL indications and cefotaxime prescriptions during two periods: pre-intervention (June 2021–May 2022) and post-intervention (June 2022–May 2023). Data was extracted from bacteriology laboratory records, hospital admissions, and pharmacy databases. The intervention consisted of staff education on VAP diagnosis, dissemination of updated literature, implementation of a standardized VAP algorithm, and active participation in daily PICU rounds.

**Figure:**
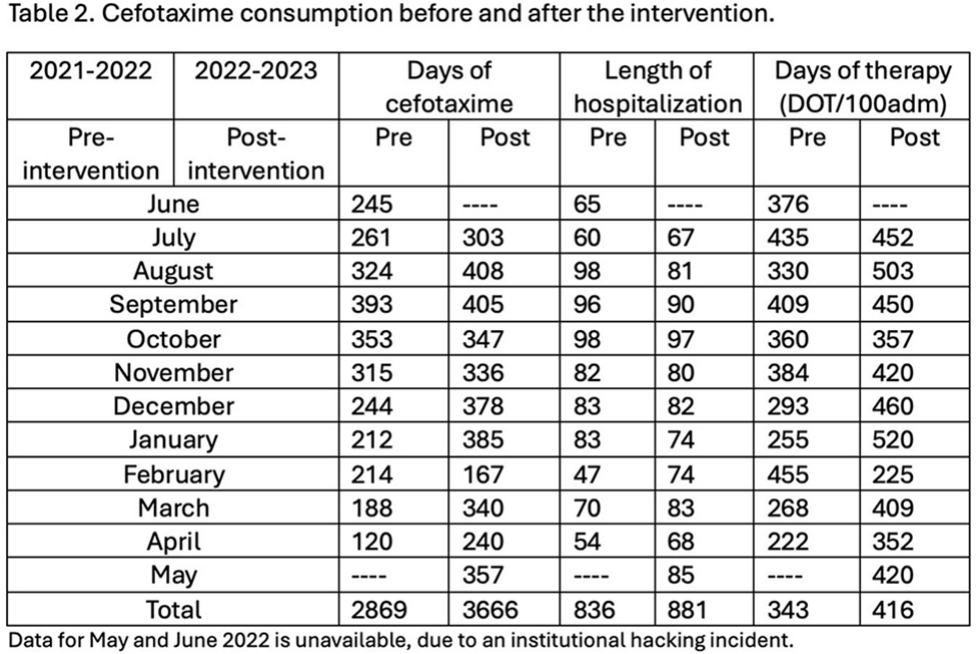

**Figure:**
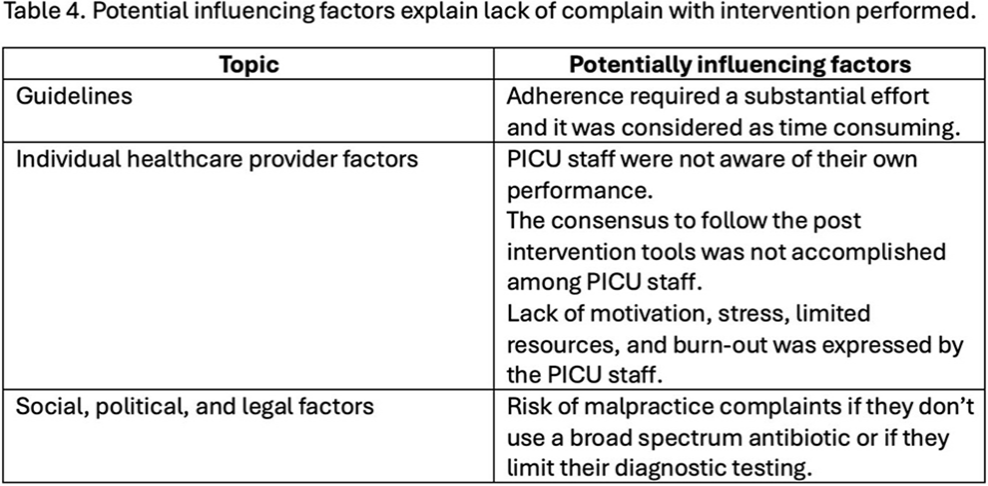

**Results:**

In the pre-intervention period, 812 BALs were performed, with a 38% positivity rate. Post-intervention, 854 BALs were performed, with a 35% positivity rate (Fig 1, 2). Cefotaxime use increased from 343 days of treatment (DOT) pre-intervention to 420 DOT post-intervention (Table 1,2). Factors potentially influencing these findings are outlined in Table 3.

**Conclusion:**

Despite targeted educational efforts and the introduction of a diagnostic algorithm, there was no reduction in the number of BALs or cefotaxime prescriptions, and the proportion of positive cultures slightly declined. These findings highlight the complexity of changing clinical practice in high-stakes environments like the PICU. Continued efforts, including real-time decision support, strengthened stewardship programs, and ongoing engagement with multidisciplinary teams, are necessary to optimize diagnostic practices and antibiotic use in pediatric VAP.

**Disclosures:**

All Authors: No reported disclosures

